# Association between bisphenols A and the risk of endometriosis: results from an updated meta-analysis

**DOI:** 10.3389/fpubh.2026.1817156

**Published:** 2026-05-04

**Authors:** Yue Chen, Junle Wu, Yuxin Wang, Fuke Wang, Qingxin Yang, Yan Zhu, Qin Zhou, Chenyu Sun, Panpan Li

**Affiliations:** 1 Department of Obstetrics and Gynecology, Zhongnan Hospital of Wuhan University, Wuhan, China; 2 Department of Clinical Medicine, The Second Clinical Medical School of Anhui Medical University, Hefei, China; 3 Department of Clinical Medicine, The First Clinical Medical School of Anhui Medical University, Hefei, China; 4 Department of Critical Care Medicine, The Third Affiliated Hospital of Kunming Medical University, Kunming, China; 5 College of Oncology, Kunming Medical University, Kunming, China; 6 Department of Obstetrics and Gynecology, Kunshan Hospital of Traditional Chinese Medicine, Kunshan, China; 7 Division of Public Health, Infectious Diseases and Occupational Medicine, Mayo Clinic, Rochester, MN, United States; 8 Department of Gynecology, Kunshan Hospital of Traditional Chinese Medicine, Kunshan, China

**Keywords:** BPA, endocrine-disrupting chemicals, endometriosis, environmental exposure, meta-analyses

## Abstract

**Background:**

Recent studies have disputed the association between bisphenol A (BPA) and endometriosis. This updated meta-analysis was conducted to investigate the impact of BPA exposure on endometriosis.

**Methods:**

This study evaluated BPA exposure and endometriosis risk, analyzing studies from Web of Science, PubMed, Embase, Cochrane Library, and Chinese databases (CNKI, CBM, Wanfang, VIP) through August 2025. Methodological quality was assessed via the Newcastle-Ottawa Scale (NOS) and the Agency for Healthcare Research and Quality Tool (AHRQ). Heterogeneity was quantified using *I*^2^ statistics. Pooled odds ratios (ORs) and 95% CIs were calculated using fixed- or random-effects models according to heterogeneity. Subgroup analysis, sensitivity analysis, and publication bias assessment were supplemented to validate the overall finding.

**Results:**

Seven studies comprising 2,488 participants were included in quantitative synthesis. The meta-analyses demonstrated that BPA exposure was associated with a statistically significant increase in endometriosis risk (OR = 1.36, 95% CI = 1.13–1.63, *p* = 0.008). Additionally, subgroup analysis indicated that BPA exposure may further elevate endometriosis risk in cases with different regions, populations, and measurement methods. Sensitivity analysis confirmed that the results of these meta-analyses were relatively robust. No publication bias was detected.

**Systematic review registration:**

https://www.crd.york.ac.uk/PROSPERO/view/CRD420251019552, identifier PROSPERO (CRD420251019552).

## 1 Introduction

Endometriosis, a chronic gynecological disorder, affects approximately 5-10% of women of reproductive age worldwide (1). Characterized by the ectopic growth of endometrial-like tissue, the condition is often accompanied by debilitating symptoms, including chronic pelvic pain, reduced fertility, and diminished quality of life (2, 3). Despite its substantial health and societal burden, the mechanisms underlying endometriosis remain poorly understood. Current theories suggest that a complex interplay of factors—such as hormonal irregularities, immune dysfunction, genetic predisposition, and environmental triggers—drives the disease. Notably, endocrine-disrupting chemicals (EDCs)—substances that interfere with hormonal systems—have emerged as potential contributors to reproductive disorders.

Bisphenol A (BPA), a synthetic compound commonly used in plastics, food containers, and industrial materials, exemplifies a well-characterized EDC (2). Exposure to BPA is ubiquitous, occurring through dietary intake, inhalation, and dermal absorption; indeed, BPA metabolites have been detected in most individuals. As a xenoestrogen, BPA disrupts endocrine function by interacting with estrogen receptors and altering androgen signaling, which may increase susceptibility to reproductive anomalies such as infertility, pregnancy-related disorders, and hormone-dependent malignancies. Although preclinical studies indicate that BPA can exacerbate endometriosis progression through estrogen-mimicking and inflammatory pathways, human epidemiological data remain inconclusive. While some observational studies (4) have associated elevated urinary BPA levels with an increased endometriosis risk, others (2, 5) have found no significant correlation—a discrepancy that may stem from variations in exposure measurement, diagnostic methods, or statistical limitations.

Previous studies have produced conflicting findings (4, 5) regarding the association between BPA exposure and endometriosis. For instance, Zhang’s (4) meta-analysis found a significant association between the risk of endometriosis and exposure to other endocrine-disrupting chemicals (including BPA), while Wu (5) reported that no positive association was observed in studying the association between plastic-related endocrine-disrupting chemicals (including BPA) and estrogen-dependent diseases in women. Since the conclusions of these two meta-analyses were contradictory due to the inclusion of incomplete studies that did not encompass all relevant recent research, their opposite findings became the focus of inquiry in this study. Although prior meta-analyses have included BPA as part of broader analyses of endocrine-disrupting chemicals, evidence specifically addressing the association between BPA exposure and endometriosis remains limited. Therefore, we conducted an updated BPA-focused meta-analysis to provide a more specific and comprehensive synthesis of the currently available evidence.

This meta-analysis systematically examines the relationship between BPA exposure and endometriosis risk by synthesizing current research and exploring heterogeneity through stratified analyses of demographic and clinical variables. By integrating updated evidence and addressing previous limitations, the study aims to enhance the epidemiological framework for understanding environmental risk factors in endometriosis, thereby guiding clinical protocols, regulatory policies, and targeted preventive strategies.

## 2 Methods

The investigation was designed and carried out in accordance with the methodological framework established by the Preferred Reporting Items for Systematic Reviews and Meta-Analyses (PRISMA) guidelines (6, 7). This approach ensured rigorous adherence to standardized protocols for systematic evidence synthesis and reporting (8). This study has been registered on the International Prospective Register of Systematic Reviews (PROSPERO). The registration ID was CRD420251019552 (9).

### 2.1 Search strategy

A systematic search was performed across four English databases (Web of Science, PubMed, Cochrane Library, Embase) and four Chinese scholarly databases (China National Knowledge Infrastructure, China Biomedical Database, Wanfang Data, VIP Database) to identify original researches examining the association between BPA and endometriosis. The search covered all records from each database’s inception through March 2026. The following search terms were used: (Bisphenol A OR BPA OR 2,2-bis(4-hydroxyphenyl)propane OR 4,4′-dihydroxy-2,2-diphenylpropane OR Diphenylolpropane OR bisphenol A, sodium salt OR bisphenol A, disodium salt) AND (endometriosis OR endometrioses OR endometrioma OR endometriomas). A supplementary review of reference lists from relevant publications was also conducted to identify additional sources. After duplicate entries were removed, a two-stage screening process was implemented: initially, titles and abstracts were evaluated for relevance, followed by a full-text review to confirm alignment with predefined inclusion criteria. To ensure consistency across databases, the English search terms were translated into corresponding Chinese terminology for use in Chinese repositories.

### 2.2 Selection criteria

The following inclusion criteria were used to select publications for analysis: (1) the study design was cross-sectional, case–control, or cohort; (2) the exposure of interest was BPA; (3) the outcome indicator was the risk of endometriosis; and (4) the analysis quantified associations between BPA exposure and endometriosis risk using reported measures of relative risk (RR), hazard ratio (HR), or odds ratio (OR) along with their respective 95% confidence intervals (CIs). Studies lacking direct statistical estimates were included if they provided datasets that allowed calculation of effect sizes. The exclusion criteria were: (1) publications with undocumented or indeterminate article classifications due to inadequate methodological reporting; (2) reviews, conference abstracts, or case reports; (3) duplicate studies; (4) non-human trials; and (5) studies that did not provide extractable data or target outcomes, lacked reliable control conditions, or failed to present sufficient data.

### 2.3 Data collection and quality assessment

To ensure data integrity and minimize bias, two independent reviewers (Yue Chen and Junle Wu) conducted study selection, data extraction, and quality assessment. All stages were mutually verified, and any discrepancies were resolved through consultation with two senior reviewers (Panpan Li and Chenyu Sun) until consensus was reached. Initial screening began with title evaluation to exclude clearly irrelevant publications, followed by sequential abstract and full-text reviews to finalize study inclusion. Data extraction was performed using a standardized template capturing parameters such as the author, publication year, study design, region, sample size, assessment method of endometriosis, study period, effect size metrics with associated 95% CIs, quality, and covariates adjusted in analytical models. Methodological quality was assessed using the Newcastle-Ottawa Scale (NOS), a validated tool for appraising non-randomized studies (10, 11). The NOS comprises eight criteria across three domains: participant selection, group comparability, and outcome/exposure ascertainment, with a maximum score of nine points; studies scoring between seven and nine were considered high quality (12). The Agency for Healthcare Research and Quality (AHRQ) tool was used to evaluate the quality of cross-sectional studies, and this instrument objectively assesses study quality across 11 categories (13). Quality scores were typically categorized into three levels: 0–3 indicating low quality, 4–7 indicating moderate quality, and 8–11 indicating high quality (14). Two authors independently assessed the quality of the studies, and any disagreements were resolved through discussion.

### 2.4 Statistical analysis

The association between BPA exposure and endometriosis risk was quantified using pooled odds ratios (ORs) with 95% confidence intervals (CIs). Analyses were conducted using Stata 15.0 (StataCorp, USA). Heterogeneity was assessed with *I*^2^ statistics; thresholds of *I*^2^ < 50% and *I*^2^ ≥ 50% guided the selection of fixed-effects or random-effects models (15), respectively. Subgroup analysis was performed by grouping studies according to geographic location, control sources, sample size, study quality, and assessment methods of endometriosis. Sensitivity analyses were performed by sequentially excluding individual studies to evaluate the stability of the results (16, 17). Publication bias was assessed via funnel plot symmetry and further evaluated with Begg’s and Egger’s tests (18, 19). Statistical significance was defined as two-tailed *p* < 0.05.

## 3 Result

### 3.1 Study characteristics

**Figure 1:**
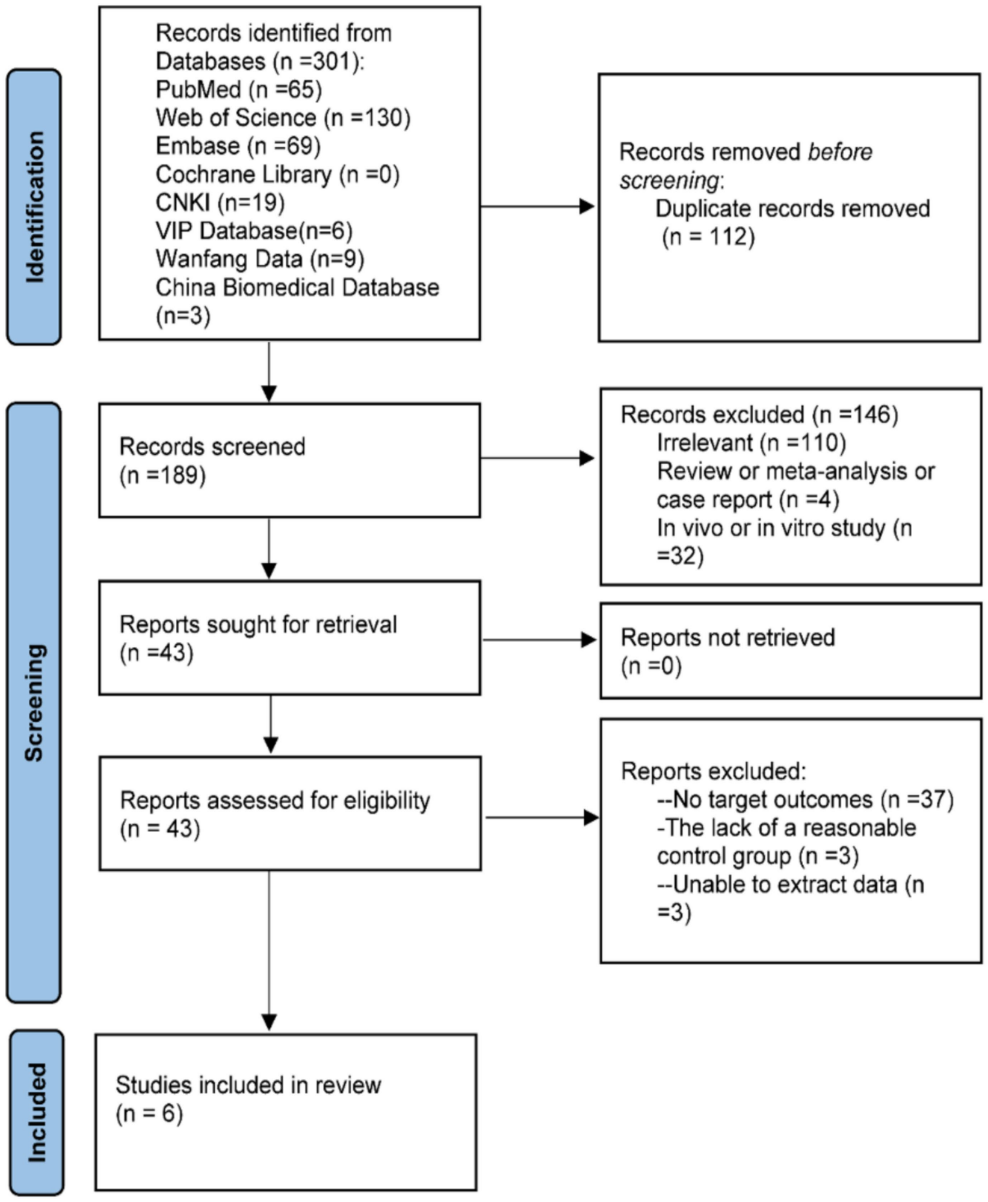
PRISMA flow diagram of included studies.

In the updated analysis, a total of 301 records were identified from databases, including PubMed (*n* = 65), Web of Science (*n* = 130), Embase (*n* = 69), CNKI (*n* = 19), VIP (*n* = 6), Wanfang (*n* = 9), and China Biomedical Database (*n* = 3), while no records were identified from the Cochrane Library. After removing duplicate records (*n* = 112), 189 records remained for title and abstract screening. Screening of titles and abstracts retained 43 articles, and a subsequent full-text evaluation resulted in 6 eligible publications (20–25) (Figure 1). The meta-analysis included 4 case–control studies (20–22, 24), one publication (25) that incorporated two cohorts, and one cross-sectional study (23), comprising a total of 2,488 participants from publications dated between 2013 and 2024. Geographically, the studies originated from the United States (20, 23, 25) (*n* = 4), Spain (22), China (24), and Iran (21) (*n* = 1 each). All investigations adjusted for confounders and were rated as either high or moderate quality. Comprehensive study details are presented in Table 1.

**Table 1:** Characteristics of studies included in this meta-analysis.

Study	Study design	Region	Sample size	Assessment method of endometriosis	Study period	OR (95% CI)	Quality	Adjustment factors
Buck et al. (25)	Cohort study	USA	473	Surgically visualized or pelvic MRI	2007–2009	0.96 (0.79, 1.19)	High	Adjusted^a^
Buck et al. (25)	Cohort study	USA	127	Surgically visualized or pelvic MRI	2007–2009	1.68 (0.96, 2.92)	High	Adjusted^a^
Upson et al. (20)	Case–control study	USA	430	Surgically confirmed	1996–2001	1.50 (0.70, 3.10)	High	Adjusted^b^
Rashidi et al. (21)	Case–control study	Iran	100	Pelvic examination and transvaginal ultrasound in lithotomic position	2013–2014	1.74 (1.40, 2.16)	High	Adjusted^c^
Peinado et al. (22)	Case–control study	Spain	124	Laparotomy or laparoscopic surgery and histological confirmation	2018–2019	1.50 (1.00, 2.30)	High	Adjusted^d^
Lee and Eata (23)	Cross-sectional study	USA	700	The reproductive health questionnaire	2003–2006	1.46 (1.00, 2.14)	Moderate	Adjusted^e^
Ao et al. (24)	Case–control study	China	534	Laparoscopic surgery	2014–2018	1.26 (1.13, 1.41)	Moderate	Adjusted^f^

a Adjusted for age, BMI, and creatinine.

b Adjusted for age, reference year, natural log-transformed urinary creatinine, education, alcohol consumption, smoking status, and race.

c Adjusted for age, BMI, parity, and education.

d Adjusted for creatinine, BMI, age, parity, and residence (rural or suburban/urban).

e Adjusted for age, race, education, and body mass index.

f Adjusted for age, BMI, education level, smoking status, alcohol consumption, and study site.

USA, United States of America.

### 3.2 Overall meta-analysis

**Figure 2:**
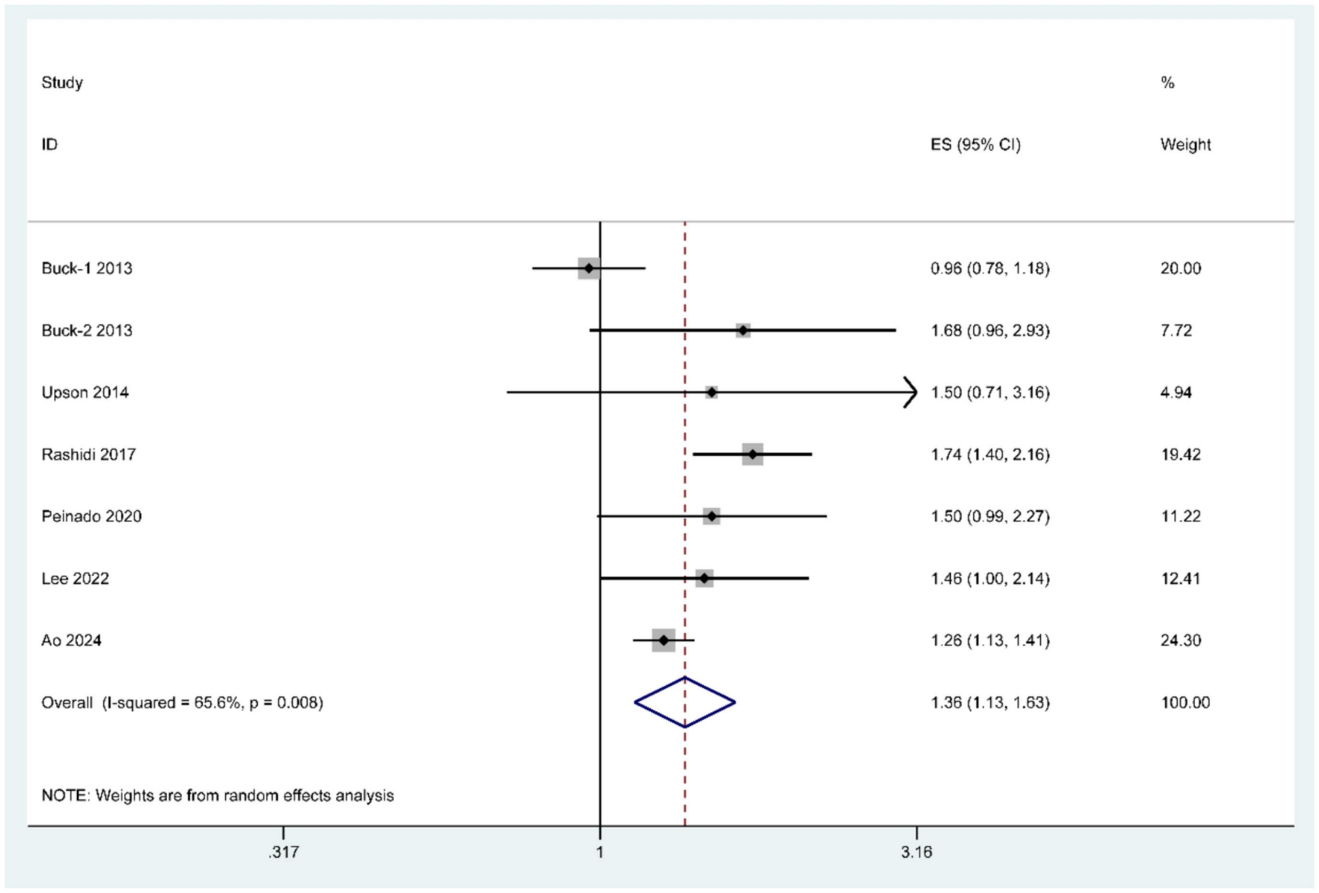
Forest plot of the association between BPA exposure and risk of endometriosis.

This meta-analysis included six articles (20–25) (comprising seven studies) that examined the association between BPA exposure and endometriosis risk. Significant heterogeneity was detected, necessitating the use of a random-effects model for the pooled analysis (Figure 2). The results demonstrated a statistically significant association between elevated BPA exposure and increased endometriosis risk (OR = 1.36, 95% CI: 1.13–1.63, P_heterogeneity_ = 0.008).

### 3.3 Subgroup analyses

Geographic stratification revealed divergent effects, with stronger associations observed in Asian countries (21, 24) (OR = 1.46, 95% CI: 1.07–2.00, *p* = 0.019) compared to Western regions (20, 22, 23, 25) (OR = 1.31, 95% CI: 1.00–1.70, *p* = 0.046). Analyses stratified by sample size demonstrated significant risks for both larger cohorts (23, 24) (>500 participants: OR = 1.41, 95% CI: 1.02–1.94, *p* = 0.036) and smaller cohorts (20–22, 25) (<500 participants: OR = 1.27, 95% CI: 1.15–1.42, *p* < 0.05). High-quality studies (20–22, 25) (OR = 1.41, 95% CI: 1.02–1.94, *p* = 0.036) and moderate-quality studies (23, 24) (OR = 1.27, 95% CI: 1.15–1.42, *p* < 0.05) all reported a significant association. Regarding the assessment of endometriosis, both surgical methods (20, 22, 24, 25) (OR = 1.23, 95% CI: 1.02–1.49, *p* = 0.029) and non-surgical testing techniques (21, 23) (OR = 1.67, 95% CI: 1.38–2.01, *p* < 0.05) have revealed a statistically significant positive association between BPA exposure and endometriosis development. Full subgroup results are detailed in Table 2.

**Table 2:** Results of the subgroup analysis.

Analysis	No. of studies	*I*^2^ (%)	P_heterogeneity_	OR (95%CI)	*p*-value	Model
Overall	7	65.6	0.008	1.36 (1.13, 1.63)	0.001	Random
Geographic location						
Western	5	51.8	0.081	1.31 (1.00, 1.70)	0.046	Random
Eastern	2	85.2	0.009	1.46 (1.07, 2.00)	0.019	Random
Sample size						
>500	2	76.1	0.002	1.41 (1.02, 1.94)	0.036	Random
<500	5	0	0.446	1.27 (1.15, 1.42)	<0.001	Fixed
Quality						
High	5	76.1	0.002	1.41 (1.02, 1.94)	0.036	Random
Moderate	2	0	0.446	1.27 (1.15, 1.42)	<0.001	Fixed
Assessment method of endometriosis						
Surgically or pelvic MRI	5	50.6	0.088	1.23 (1.02, 1.49)	0.029	Random
Non-surgical operation	2	0	0.432	1.67 (1.38, 2.01)	<0.001	Fixed

### 3.4 Sensitivity analyses and publication bias

**Figure 3:**
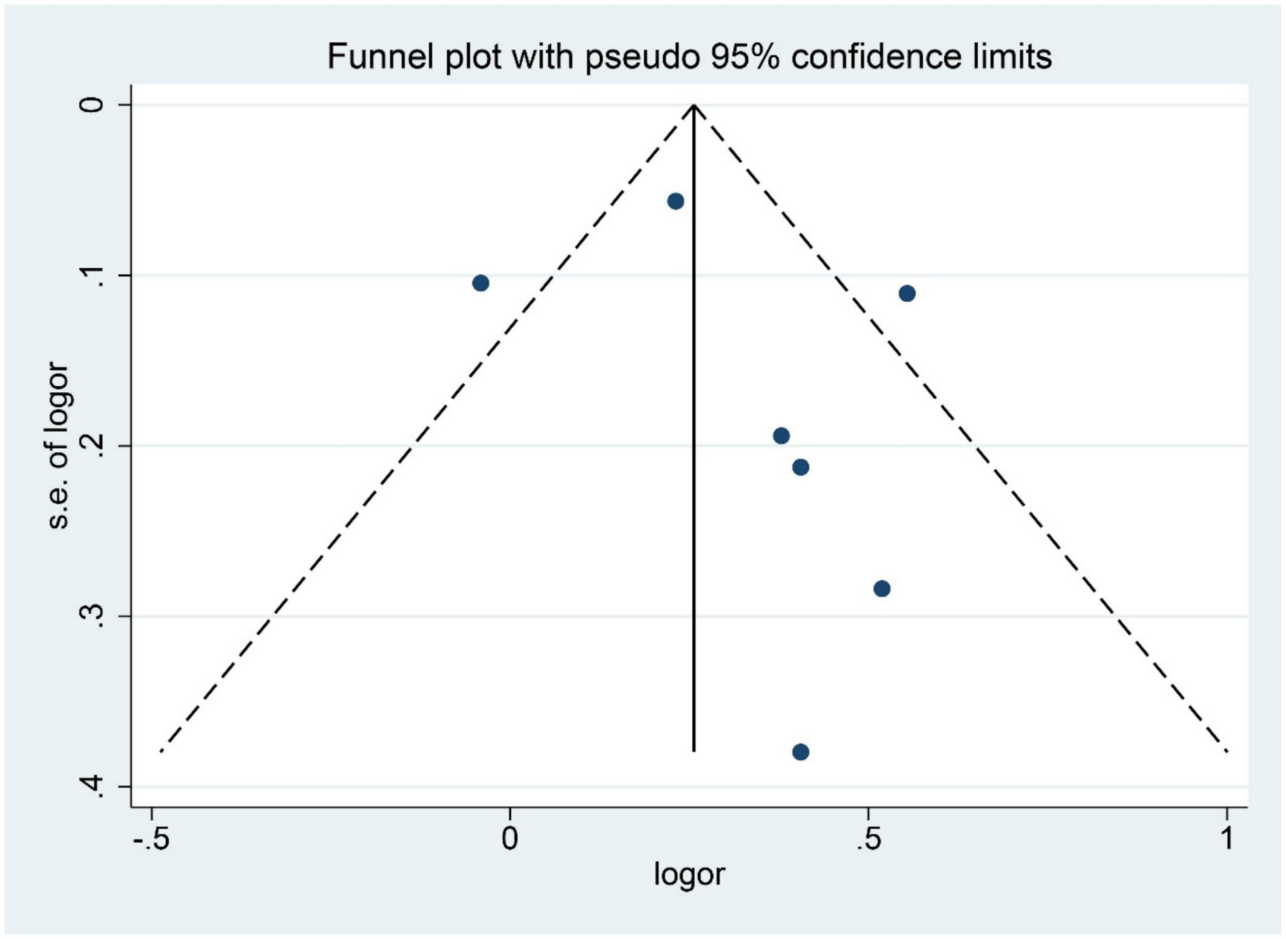
Funnel plot of the association between BPA exposure and risk of endometriosis.

Sensitivity analysis, conducted using the leave-one-out approach, revealed consistent effect estimates, confirming the robustness of the primary findings. The results remained consistent when switching between fixed-effect and random-effects models. Visual inspection of the funnel plot did not suggest marked asymmetry (Figure 3). In addition, Begg’s test (*Z* = 0.60, *p* = 0.548) and Egger’s test (*t* = 0.81, *p* = 0.454) did not indicate significant publication bias. However, because only seven datasets were included in the quantitative synthesis, these methods had limited statistical power to detect publication bias, and the results should therefore be interpreted with caution.

## 4 Discussion

The association between BPA exposure and endometriosis remains controversial in the existing literature. Compared with previous meta-analyses (4, 5), the present study provides an updated and more focused quantitative synthesis of this association. Rather than examining BPA only as part of a broader group of endocrine-disrupting chemicals, this meta-analysis specifically evaluates the relationship between BPA exposure and endometriosis. In addition, we incorporated more recent eligible evidence, including the study by Ao et al. (24), and included the study by Lee and Eata (23), which was not considered in the previous meta-analysis. We also performed more detailed subgroup analyses according to country, participant source, sample size, diagnostic method, study quality, and confounder adjustment. Therefore, the added value of the present study lies not only in updating the available evidence but also in providing a more focused and methodologically detailed assessment of the consistency of the observed association. Based on the pooled results, BPA exposure may be associated with an increased risk of endometriosis.

Subgroup analyses showed that BPA exposure was associated with an increased risk of endometriosis across various settings, including different countries, hospital-based and population-based studies, populations of different sizes, and different diagnostic methods. Specifically, significant associations were observed in both Western countries and Eastern countries, suggesting that the observed relationship was not restricted to a specific geographic region. Similarly, the association persisted in studies with both larger sample sizes and smaller sample sizes, indicating that the overall finding was not driven solely by study size. When stratified by study quality, statistically significant associations were found in both high-quality and moderate-quality studies, which further supports the robustness of the pooled result. Besides, a positive association was observed in both surgically confirmed and non-surgically defined endometriosis, although the pooled estimate was larger in the non-surgical subgroup. Rather than indicating a stronger biological effect, this discrepancy may be related to differences in diagnostic criteria across studies. In particular, variation in diagnostic specificity and the possibility of outcome misclassification in non-surgical assessments may have contributed to the difference in effect estimates. Overall, these subgroup findings suggest that the association between BPA exposure and endometriosis is relatively stable across regions, study sizes, study quality, and diagnostic approaches, thereby strengthening the evidence that BPA may be involved in the development of endometriosis.

Several biological mechanisms may underlie the observed association between BPA exposure and an increased risk of endometriosis. At the molecular level, BPA is well known to act as an endocrine disruptor with estrogen-like activity (26). By binding to estrogen receptors, including ERα and ERβ, BPA can activate both genomic and non-genomic signaling pathways, thereby disrupting hormonal homeostasis in the female reproductive tract (27, 28). Experimental studies in animal models have further shown that prenatal BPA exposure may induce endometriosis-like lesions through dysregulation of key genes involved in progesterone signaling, particularly PGR and its downstream target Hand2. This may contribute to progesterone resistance, which is considered a hallmark of endometriosis (28, 29). In addition to its endocrine-disrupting effects, BPA exposure has also been linked to increased oxidative stress and inflammatory responses (30). Elevated reactive oxygen species (ROS) levels can damage cells and promote a pro-inflammatory environment that facilitates the adhesion and proliferation of ectopic endometrial cells. Emerging evidence also suggests that epigenetic alterations may play an important role in mediating the effects of BPA on the endometrium (31). Abnormal DNA methylation patterns in regulatory genes have been reported in both animal and *in vitro* studies, raising the possibility that BPA exposure during critical developmental windows may induce long-lasting changes in gene expression (32). Such epigenetic modifications could explain the transgenerational effects observed in some studies (33), where offspring of BPA-exposed mothers show increased susceptibility to endometriosis and other reproductive disorders later in life. Moreover, BPA may contribute to the progression of endometriosis by aggravating immune and inflammatory dysfunction (34). As an inflammatory disease, endometriosis is characterized by an aberrant immune response in the peritoneal cavity (35, 36). BPA may increase the secretion of pro-inflammatory cytokines and chemokines, thereby altering the local immune environment. This inflammatory milieu can hinder the clearance of retrograde menstrual debris and promote the survival and implantation of endometrial cells at ectopic sites. In parallel, BPA-induced oxidative stress may further promote DNA damage, aberrant cell proliferation, and angiogenesis, all of which are critical processes in the establishment and progression of endometriotic lesions (34).

Several limitations of this meta-analysis should be noted. First, subgroup analyses by endometriosis localization were not feasible, leaving the relationship between BPA exposure and the specific locations of endometriotic lesions unclear. Second, because all included studies were observational, a definitive causal relationship cannot be established. Third, the number of included studies was relatively small, which may have limited the statistical power of the analysis and reduced the generalizability of the findings. Finally, the definition of endometriosis was not entirely consistent across the included studies, as different diagnostic approaches were used, including surgical confirmation, imaging-based assessment, and questionnaire-based ascertainment. This variation may have introduced heterogeneity in outcome ascertainment and should be taken into consideration when interpreting the pooled results. Notwithstanding these limitations, our meta-analysis has several strengths. First, compared with previous meta-analyses, our study applied more rigorous inclusion criteria, which helped ensure the overall quality of the included evidence (4, 5). Second, we performed subgroup analyses according to study characteristics, including geographic region, exposure assessment period, study design, sample size, and adjustment for confounding factors. Finally, the pooled effect estimates were primarily based on multivariable-adjusted ORs reported in the original studies, which improved the reliability of the overall results.

This updated meta-analysis suggests a possible association between BPA exposure and an increased risk of endometriosis. However, given the limited number of included studies and the observational design of the available data, the results should be interpreted cautiously. Further large-scale and well-designed studies are needed to confirm this association and to better understand the potential role of BPA in the development of endometriosis.

## Data Availability

Publicly available datasets were analyzed in this study. This data can be found at: https://pubmed.ncbi.nlm.nih.gov/.
